# An Early Presenting Esophageal Schwannoma

**DOI:** 10.1155/2011/165120

**Published:** 2011-05-22

**Authors:** Shelly S. Choo, Maurice Smith, Ashley Cimino-Mathews, Stephen C. Yang

**Affiliations:** ^1^Division of Thoracic Surgery, Department of Surgery, The Johns Hopkins University, Baltimore, MD 21205, USA; ^2^Department of Pathology, The Johns Hopkins University, Baltimore, MD 21205, USA

## Abstract

Esophageal schwannoma is a rare diagnosis and historically has been a tumor of middle-aged females. We report a case of a 22-year-old male presenting initially with dyspnea secondary to tracheal compression from an 8 × 6 × 3.0 cm esophageal schwannoma. The tumor was surgically resected, and diagnosis was confirmed with immunohistochemical and pathological studies. We report the youngest case of esophageal schwannoma in an otherwise healthy individual.

## 1. Introduction

Schwannomas of the esophagus are peripheral nerve sheath tumors characterized by peripheral lymphoid cuffing, benign nuclear atypia, and spindle-shaped cells [1]. We report a case of a large benign esophageal schwannoma causing symptoms of initial dyspnea and later progressing to dysphagia.

## 2. Case Report

**Figure 1:**
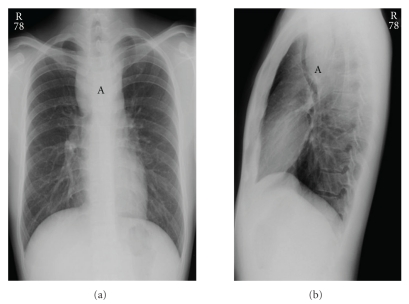
Chest X-ray AP (a) and lateral (b) views showing a large mediastinal mass (A).

**Figure 2:**
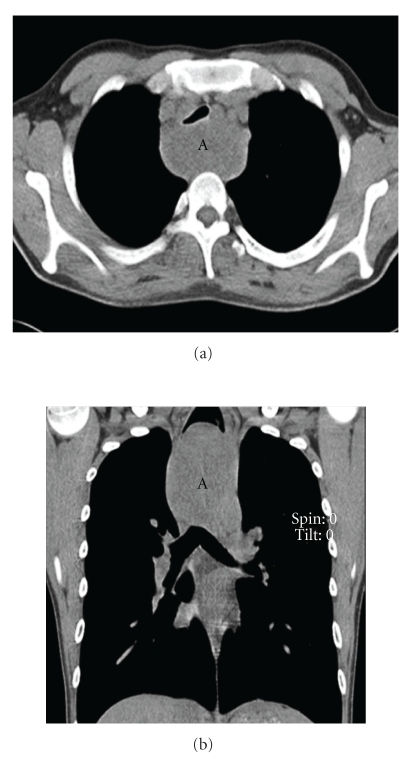
CT scan (a) with coronal view (b) showing large posterior mediastinal mass (A) with tracheal compression.

A 22-year-old Asian American male presented for symptoms of dyspnea for six months that were being treated as asthma. This progressed to solid food dysphagia and a 20-pound weight loss over two years. The remainder of his history was noncontributory, and the physical exam was only positive for audible wheezing. A chest X-ray revealed a large mediastinal mass, with subsequent computed tomography (CT) scan demonstrating an upper esophageal mass that adhered to the posterior wall of the esophagus. The trachea, significantly narrowed, was seen to be bowing anteriorly (Figures 1 and 2). 

Flexible esophagoscopy identified the mass at 19 cm from the incisors and with attachment to the left anterolateral esophageal wall. The following week, he was taken to the operating room for excision of the mass.

A right posterolateral thoracotomy was performed. The tumor was found in the submucosal layer along the wall. The azygos vein was visualized inferior to the mass and doubly ligated. The vagus nerve was free from involvement. The esophagus was opened from below the thoracic inlet to the level of the azygos vein. The mass was dissected and easily shelled out. Areas of the posterior wall were resected for tumor margins, and the esophagotomy was closed in two layers.

**Figure 3:**
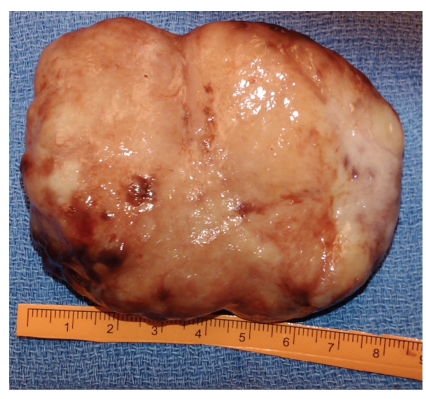
The enucleated esophageal schwannoma was yellowish-white, smooth, and well-defined without any pedunculated portions.

**Figure 4:**
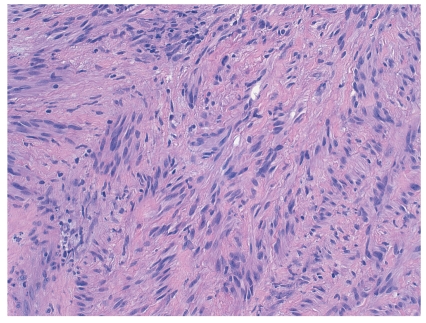
Histology of the esophageal mass upon resection. Microscopic examination of the esophageal mass revealed palisading spindled cells with dense chromatin, rare mitoses and scattered atypia (H&E, x100). Immunohistochemistry revealed the tumor to be positive for the S100 protein (not shown), confirming nerve sheath origin.

Gross examination of the esophageal mass revealed a smooth, multilobulated, but well-circumscribed mass measuring 8.5 cm in greatest dimension (Figure 3). On cut section, the mass was uniform and tan with no discrete areas of hemorrhage or necrosis. Histologic examination of the mass revealed palisading spindled cells with scattered atypia, dense chromatin, and rare mitoses (Figure 4) in a background of prominent hyalinized vessels and a lymphoplasmacytic infiltrate. Immunohistochemical studies revealed the tumor to be positive for the S100 protein, confirming nerve sheath origin; the tumor was negative for actin, desmin, CD117 (ckit), and alk-1.

The patient tolerated the procedure well. His postoperative course was complicated by a contained leak at the esophagotomy repair site that was managed conservatively and postoperative pneumonia. He remained afebrile and hemodynamically stable. He was discharged postoperative day eight with a regular diet. At the three month followup visit, he was eating normally and had no symptoms.

## 3. Discussion

Esophageal schwannomas are rare with only 30 reported cases in the English literature with many coming from Asia (18/27) [2–4]. Prior to our report, the youngest case reported in an otherwise healthy individual was age 29 [5]. The majority of cases present with dysphagia, but dyspnea has been documented in three other cases all in younger patients (ages 29, 30, and 32).

Schwannomas are generally treated via surgical resection, usually through a thoracotomy with enucleation. With complete excision, the prognosis of schwannomas is generally excellent with recurrence being rare [5]. 

Our case is the youngest in an otherwise healthy individual and one of the largest to present with an esophageal schwannoma. His Asian decent underscores the predominance of this disease in this population. Though rare, or perhaps underreported in the medical literature, it should be on the differential for unexplained dyspnea or dysphagia in any age group when radiographic text reveals a large mediastinal mass.
